# S100A8 and S100A9: DAMPs at the Crossroads between Innate Immunity, Traditional Risk Factors, and Cardiovascular Disease

**DOI:** 10.1155/2013/828354

**Published:** 2013-12-22

**Authors:** Alexandru Schiopu, Ovidiu S. Cotoi

**Affiliations:** ^1^Department of Clinical Sciences, Lund University Malmö, 205 02 Malmö, Sweden; ^2^Cardiology Clinic, Skane University Hospital Malmö, Inga Marie Nilssons gata 46, Floor 2, 205 02 Malmö, Sweden; ^3^Department of Cellular and Molecular Biology, University of Medicine and Pharmacy of Tîrgu Mureş, 540139 Tîrgu Mureş, Romania

## Abstract

Amplification of innate immune responses by endogenous danger-associated molecular patterns (DAMPs) promotes inflammation. The involvement of S100A8 and S100A9, DAMPs belonging to the S100 calgranulin family, in the pathogenesis of cardiovascular disease is attracting an increasing amount of interest. S100A8 and S100A9 (also termed MRP8 and MRP14) preferentially form the S100A8/A9 heterodimer (MRP8/14 or calprotectin) and are constitutively expressed in myeloid cells. The levels of circulating S100A8/A9 in humans strongly correlate to blood neutrophil counts and are increased by traditional cardiovascular risk factors such as smoking, obesity, hyperglycemia, and dyslipidemia. S100A8/A9 is an endogenous ligand of toll-like receptor 4 (TLR4) and of the receptor for advanced glycation end products (RAGE) and has been shown to promote atherogenesis in mice. In humans, S100A8/A9 correlates with the extent of coronary and carotid atherosclerosis and with a vulnerable plaque phenotype. S100A8/A9 is locally released following myocardial infarction and amplifies the inflammatory responses associated with myocardial ischemia/reperfusion injury. Elevated plasma levels of S100A8/A9 are associated with increased risk of future coronary events in healthy individuals and in myocardial infarction survivors. Thus, S100A8/A9 might represent a useful biomarker and therapeutic target in cardiovascular disease. Importantly, S100A8/A9 blockers have been developed and are approved for clinical testing.

## 1. Introduction

Inflammation plays a central role in the development of atherosclerosis and in plaque vulnerability [1]. The chronic, low-grade inflammatory process characteristic of atherosclerosis development in the arterial wall is sustained by a constant interplay between innate and adaptive immunity [2]. The primary function of the innate immune system is to combat pathogen invasion, but it can also be activated by endogenous ligands under conditions of immunological stress [3]. Neutrophils and monocytes, central components of innate immunity, express pattern recognition receptors (PRRs) on their surface that bind evolutionarily conserved structures such as bacterial pathogen-associated molecular patterns (PAMPs) and endogenous danger-associated molecular patterns (DAMPs), leading to cell activation [3]. DAMPs, also known as alarmins, are intracellular molecules that involved in cellular function under normal homeostasis, which are released after cell death, signaling tissue damage [3, 4].

The S100 proteins form a calcium-binding cytosolic protein family defined by their common ability to dissolve in 100% ammonium sulphate [5]. Several S100 proteins have so far been identified as DAMPs, including S100A7 [6], S100A8, S100A9, S100A12 [5, 7], and S100A15 [6]. S100A8, S100A9, and S100A12 are produced by cells of myeloid origin [8] and have been linked with cardiovascular disease (CVD) [9, 10]. Clinical data show clear correlations between S100A12 and the severity of coronary and carotid atherosclerosis [10–12], but mechanistic studies on the role of S100A12 in CVD are hampered by the absence of this protein in mice. The present review will attempt to summarize the increasing body of evidence demonstrating the involvement of S100A8 and S100A9 in atherogenesis, plaque vulnerability, myocardial infarction (MI), and heart failure.

S100A8 and S100A9 are also known as calgranulins A and B or myeloid-related proteins (MRP) 8 and 14. S100A8 and S100A9 are constitutively expressed in neutrophils, monocytes [8], and dendritic cells [13] but can also be induced upon activation in other cell types such as mature macrophages [14–16], vascular endothelial cells [17–19], fibroblasts [20], and keratinocytes [21]. In neutrophils, S100A8 and S100A9 constitute ~45% of all cytosolic proteins, compared to only about 1% in monocytes [8]. S100A8 expression seems to differ between subsets of human monocytes, as higher levels of S100A8 mRNA were detected in classical CD14^++^CD16^−^ monocytes compared to their nonclassical CD14^+^CD16^++^ counterparts [22]. S100A8 and S100A9 exist as homodimers but preferentially form the S100A8/A9 heterodimer (also called calprotectin) in the presence of Zn^2+^ and Ca^2+^. Intracellularly, S100A8/A9 promotes phagocyte migration by promoting tubulin polymerization and stabilization of tubulin microfilaments in a calcium dependent manner [23].

Extracellular S100A8/A9 is primarily released from activated or necrotic neutrophils and monocytes/macrophages and is involved as an innate immune mediator in the pathogenesis of various diseases with an inflammatory component [24, 25]. We have recently studied the correlations between S100A8/A9 and the circulating numbers of neutrophils, lymphocytes, platelets, total monocytes, and different monocyte subpopulation in human blood. Our data suggests that neutrophils seem to be the main source of systemic S100A8/A9, as neutrophils were the only cell population that strongly and independently correlated with plasma S100A8/A9 levels [26]. Interestingly, both pro- and anti-inflammatory functions of S100A8, S100A9, and S100A8/A9 have been reported, suggesting that the functions of S100A8/A9 might be concentration-dependent and influenced by the cellular and biochemical composition of the local milieu [27]. S100A8, S100A9, and S100A8/A9 promote neutrophil and monocyte recruitment by activating the microvascular endothelium [28] and by stimulating phagocyte Mac-1 expression, affinity and binding to ICAM-1, fibronectin, and fibrinogen [29–32]. However, other authors failed to reproduce the chemotactic activity of S100A8 and S100A9 and demonstrate instead a fugitactic (repellent) effect on neutrophils at picomolar concentrations, which may contribute to resolution of inflammation and tissue repair [33, 34]. Oxidant scavenging [35], matrix metalloproteinase (MMP) inhibition by Zn^2+^ chelation [36] and inhibition of reactive oxygen species production in phagocytes [37–39] are additional anti-inflammatory and tissue protective mechanisms that were proposed for S100A8, S100A9 and S100A8/A9.

The toll-like receptor 4 (TLR4) and the receptor for advanced glycation endproducts (RAGE) have so far been suggested as innate immune receptors of S100A8/A9 [40–42]. S100A8/A9 binding triggers MyD88-mediated TLR4 signaling, leading to NF-kB activation and secretion of pro-inflammatory cytokines such as TNF*α* and IL-17 [40, 43, 44]. The S100A8/A9-TLR4 interaction has been shown to be involved in the pathogenesis of systemic infections, autoimmune diseases, malignancy, and acute coronary syndrome [40, 43, 45–48]. Similarly, S100A8/A9 binding to RAGE leads to MAP kinase phosphorylation and NF-kB activation, promoting leukocyte production in the bone marrow [49], carcinogenesis [50–52], cardiomyocyte dysfunction [53] and postischemic heart failure [54]. RAGE activation by S100A8/A9 or other ligands leads to further enhancement of S100A8/A9 production, creating a putative positive feedback loop in chronic inflammation [55, 56]. Interestingly, it has recently been shown that, in contrast to neutrophils, S100A9-defficient dendritic cells secrete increased amounts of inflammatory cytokines upon TLR4 stimulation, suggesting that S100A9 might function as an innate immune suppressor in this particular cell population [13].

S100A8/A9 binds heparan sulphate proteoglycans and carboxylated glycans on endothelial cells [57, 58] and triggers endothelial activation, characterized by enhanced production of inflammatory cytokines and chemokines [28, 56], increased expression of adhesion molecules [28, 56], and increased platelet aggregation at the surface of the endothelium [28]. Additionally, endothelial cells treated with S100A8/A9 were shown to downregulate antiapoptotic genes and genes responsible for the integrity of the endovascular monolayer [28, 59]. Extended S100A8/A9 exposure leads to endothelial cell dysfunction and increased endothelial permeability [59]. These effects are partly mediated by RAGE [41] and exacerbated by hyperglycemia [56, 60].

Oxidative modifications of S100A8 and S100A9 induced by reactive oxygen species mainly target cysteine and methionine residues and have been shown to regulate function. The different reversible and irreversible oxidative modifications of S100 proteins described to date and their potential functional consequences have been expertly reviewed elsewhere [27, 61]. Oxidation of methionines 63 and 83 on S100A9 and of cysteine 42 on S100A8 inhibits both the chemotactic and the repellent effects of the proteins on neutrophils, whereas the oxidation-resistant mutants were shown to retain function [33, 34]. Conversely, oxidation of these residues was found to be required for the antifungal activities of S100A8/A9 [62]. HOCl induced oxidation of S100A8 and S100A9 generates stable cross-linked dimers, trimmers, and S100A8-S100A9 complexes of different sizes that were found in human carotid plaques [18]. Oxidized S100A8 was also found to predominate in sputum from asthmatic patients compared to native S100A8 [35], suggesting that these mechanisms are involved *in vivo* in the pathogenesis of inflammatory disease in humans. S100A8 and S100A9 are much more sensitive to oxidation compared to low-density lipoproteins and albumin and the authors propose that the high amounts of S100 proteins present in atherosclerotic plaques might contribute to oxidant scavenging and protect other proteins and tissue components from oxidative damage during inflammation [18]. Interestingly, S100A9 is less susceptible to oxidation compared to S100A8 [18] and has a much higher affinity for TLR4 and RAGE compared to S100A8 and S100A8/A9 [42]. It is tempting to speculate that under mild oxidative conditions, S100A8/A9 oxidation releases S100A9 from the heterocomplex, leading to TLR4 and RAGE binding and activation. This hypothesis would explain the lack of widespread receptor activation under steady-state physiological conditions despite the presence of large amounts of circulating S100A8/A9. However, other authors propose S100A8 to be the main active component of the S100A8/A9 complex [40], so this issue remains controversial. The influence of S100A8/A9 oxidation on TLR4 and RAGE binding and activation has not been investigated and it needs further clarification.

## 2. S100A8/A9 and Cardiovascular (CV) Risk Factors

**Figure 1:**
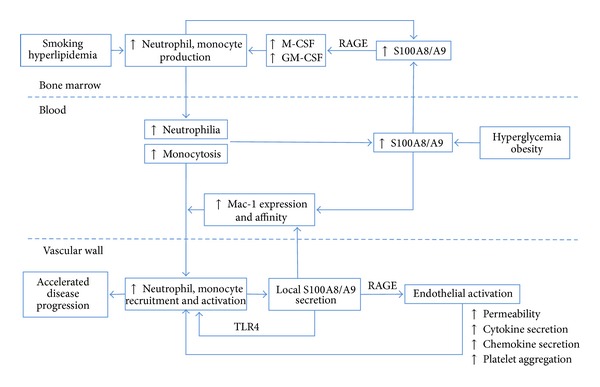
Overview of the interplay between S100A8/A9, traditional CV risk factors, circulating phagocytes, and atherogenesis. Smoking, hyperlipidemia, hyperglycemia, and obesity induce elevated S100A8/A9 production either directly or indirectly by stimulating neutrophilia and monocytosis. S100A8/A9 enhances phagocyte production in the bone marrow and facilitates their recruitment into the vascular wall through endothelial activation and increased Mac-1 expression and affinity. These effects are primarily mediated by RAGE and accelerated by hyperglycemia. In the vascular wall, S100A8/A9 binding to TLR4 triggers phagocyte activation and secretion of inflammatory cytokines, further contributing to phagocyte recruitment and accelerated atherogenesis. M-CSF: macrophage colony stimulating factor; GM-CSF: granulocyte-macrophage colony stimulating factor; RAGE: receptor for advanced glycation end products; TLR4: toll-like receptor 4.

Diabetes mellitus, obesity, smoking, and hyperlipidemia are traditional CV risk factors that have been associated with increased levels of S100A8/A9 in plasma. An overview of the interplay between S100A8/A9, traditional CV risk factors, circulating phagocytes, and vascular inflammation is presented in Figure 1. Hyperglycemia induces the production of reactive oxygen species (ROS) in human endothelial cells *in vitro* and in aortic endothelial cells of diabetic mice *in vivo*, leading to overexpression of S100A8 and RAGE [17]. Similarly, hyperglycemia-induced expression of ROS in neutrophils leads to increased S100A8/A9 secretion [49]. S100A8/A9 binds RAGE on common myeloid progenitors and macrophages in the bone marrow and stimulates production of growth factors, leading to accelerated myelopoiesis and increased release of neutrophils and inflammatory Ly6C^hi^ monocytes into the circulation [49]. As a result, hyperglycemic diabetic mice have higher concentrations of S100A8/A9 in plasma and increased numbers of circulating leukocytes. This phenotype can be reversed by pharmacological reduction of systemic glucose levels or by knocking out the RAGE receptor [49]. In diabetic LDLR^−/−^ mice, accelerated recruitment of Ly-6C^hi^ monocytes into the atherosclerotic plaques leads to impaired lesion regression, which might explain the increased severity of atherosclerosis found in diabetic patients [49]. Neutrophil recruitment into the arterial wall was not assessed in this study. These experimental results are supported by clinical data demonstrating elevated S100A8/A9 levels in patients with type 2 diabetes or impaired glucose tolerance compared with nondiabetic controls [63]. Additionally, plasma S100A8/A9 was found to positively correlate with measures of impaired glucose metabolism such as insulin resistance, fasting blood glucose [63], and glycosylated hemoglobin A1c (HbA1c) [26].

Body-mass index (BMI) is an independent determinant of S100A8/A9 concentrations [26, 63]. Among nondiabetics, plasma S100A8/A9 was found to be higher in obese versus non-obese individuals [63–65]. This effect could not be observed in diabetic subjects [63, 64], suggesting the presence of partially overlapping mechanisms responsible for increased production of S100A8/A9 in obesity and diabetes. Weight loss in obese nondiabetic subjects leads to significantly decreased S100A8/A9 alongside insulin resistance and plasma lipids [63]. Interestingly, the reduction in circulating S100A8/A9 levels was not associated with lower blood leukocyte counts, suggesting that obesity is associated with increased leukocyte activation and S100A8/A9 production rather than increased leukocytosis [63].

As previously discussed, neutrophils seem to be the main source of circulating S100A8/A9 [26] and blood neutrophil counts correlate strongly with plasma S100A8/A9 concentrations [26, 63]. Smoking and hyperlipidemia stimulate granulopoiesis and S100A8/A9 production. Active smoking is a strong stimulus for neutrophilia in apparently healthy individuals [66] and smokers have elevated S100A8/A9 levels [26]. Similarly, hyperlipidemia stimulates neutrophilia through increased granulopoiesis and enhanced neutrophil release from the bone marrow [67]. Our own observations in a cohort of apparently healthy individuals show that LDL positively and HDL negatively influence plasma S100A8/A9 concentration independently of BMI, smoking, and glycemic control [26]. Thus, several traditional cardiovascular risk factors increase systemic S100A8/A9 levels either directly by phagocyte activation and S100A8/A9 release or indirectly by stimulation of neutrophil and monocyte production in the bone marrow.

## 3. S100A8/A9 and Atherosclerosis 

S100A8/A9 is an active mediator in the pathogenesis of various autoimmune and inflammatory conditions [24, 25]. In recent years, the involvement of neutrophils and S100A8/A9 in CV disease (CVD) has attracted an increasing amount of interest [9]. S100A8/A9 is thought to accelerate atherogenesis through increased recruitment and activation of neutrophils and monocytes in the arterial wall (Figure 1). Despite early controversy, the proatherogenic role of neutrophils, the main source of circulating S100A8/A9, is now firmly established [67–70]. S100A8 and S100A9 are present in atherosclerotic plaques in both mice and humans (Table 1) [18, 71–73] and S100A8/A9 has been proposed as a potential target for plaque-directed accumulation of gadolinium nanoprobes in imaging and therapeutic applications [71]. Signaling through TLR4 and RAGE, the endogenous receptors of S100A8/A9 have been shown to be proatherogenic. Plaque size is reduced in atherosclerotic mice deficient in TLR4 or its adaptor protein MyD88 [74, 75] and RAGE deficiency is associated with delayed plaque progression and attenuated vascular inflammation in hyperlipidemic ApoE^−/−^ mice [41]. RAGE is overexpressed in atherosclerotic plaques collected from diabetic patients and from mice rendered diabetic by streptozotocin treatment [76, 77]. The diabetic ApoE^−/−^ mice have elevated plasma S100A8/A9 levels and develop larger atherosclerotic lesions characterized by increased content of S100A8/A9, advanced glycation endproducts (AGEs), activated NF-kB, VCAM-1, and MCP-1 [77]. These effects were abrogated in the absence of RAGE [77], suggesting that RAGE and its ligands play important roles in the accelerated atherogenesis associated with diabetes.

The S100A9^−/−^ mouse strain has facilitated important advances in the understanding of the role of S100A8/A9 in myeloid cell function and in vascular disease [78]. S100A8 is unstable in the absence of S100A9, so these mice lack both S100A8 and S100A9 proteins [78]. S100A8 mice are not viable [79]. It has been shown that S100A9 deficiency impairs the migratory capacity and cytokine production of neutrophils and monocytes/macrophages [13, 23, 80–82]. Leukocyte recruitment and lesion size were significantly reduced in S100A9^−/−^ mice undergoing femoral artery wire injury [82]. The hyperlipidemic ApoE^−/−^S100A9^−/−^ double knockout mice develop smaller atherosclerotic lesions with lower macrophage infiltration compared to their ApoE^−/−^ counterparts [82]. Unexpectedly, atherosclerosis development was not delayed in hyperlipidemic LDLR^−/−^ mice reconstituted with S100A9^−/−^ bone marrow, suggesting that local S100A9 expression in nonmyeloid cells might play an important role [13]. In an attempt to explain these contrasting findings, the authors have found opposite effects of S100A9 deficiency in neutrophils and dendritic cells. While S100A9^−/−^ neutrophils secreted markedly less TNF*α* and MCP-1 in response to LPS stimulation, inflammatory cytokine production in dendritic cells was exacerbated by S100A9 deficiency and exogenous S100A8/A9 was shown to inhibit the ability of activated DCs to induce T cell proliferation *in vitro *[13].

The link between S100A8/A9 and atherosclerosis is further supported by clinical studies demonstrating a positive relationship between plasma S100A8/A9 and the severity of coronary artery disease (CAD) in type 1 and type 2 diabetic patients (Table 1) [49, 83]. S100A8/A9 was also shown to correlate with carotid intima-media thickness (IMT) in a small subgroup of diabetic patients without CAD [84] and in middle-aged individuals with no previous history of CVD [26]. Circulating neutrophil numbers presented similar associations with carotid IMT, independently of traditional CV risk factors [26]. Detailed immunohistochemical and biochemical analysis of human carotid plaques have demonstrated an increased amount of S100A8/A9 in vulnerable lesions characterized by large lipid cores, intense macrophage infiltration, low collagen content, and elevated concentrations of inflammatory cytokines and matrix metalloproteinases [72]. The authors found an increased number of S100A8 and S100A9 positive macrophages in rupture-prone atheromas [72], consistent with experimental data showing that S100A9 positive monocytes are preferentially recruited into atherosclerotic plaques [73]. Ultrasound analysis of carotid plaques in type 2 diabetic patients demonstrated that the presence of echolucent plaques, generally considered to belong to the vulnerable phenotype, is associated with increased plasma levels of S100A8/A9 [85]. In patients undergoing carotid endarterectomy, high concentrations of S100A8/A9 in plasma and in the carotid plaques were associated with the incidence of acute CV events (fatal or nonfatal) during follow-up, independently of the classic CV risk factors and CRP [86]. Associations between plasma S100A8/A9 and CV risk have also been found to be valid in healthy individuals and in systemic lupus erythematosus (SLE) patients. Healy et al. reported that apparently healthy postmenopausal women with S100A8/A9 levels within the highest quartile run a 3.8 times higher risk to develop acute CV events during a median follow-up period of 3 years, independently of other CV risk factors [87]. Recently published data from our group demonstrate that the independent associations between elevated S100A8/A9 in apparently healthy women and the incidence of coronary events and CV death are paralleled by similar associations for circulating neutrophil numbers [26]. SLE is a chronic inflammatory disease associated with increased CV risk [88]. Serum S100A8/A9 was found to be elevated in patients with clinically inactive SLE and prevalent CVD [89], but it remains to be determined whether S100A8/A9 can predict incident CV events in this population in prospective studies.

## 4. S100A8/A9 in Acute Coronary Syndrome

The demonstrated associations between S100A8/A9 and the incidence of acute CV events have prompted further research into the role of S100A8/A9 as potential disease mediator and prognostic biomarker in coronary artery disease. Plasma S100A8/A9 was found to be highly increased during the ischemic event in acute coronary syndrome patients compared with stable angina or with individuals with angiographically assessed normal coronary artery morphology (Table 1) [90]. As cardiac myocytes subjected to ischemia do not upregulate S100A8 and S100A9 mRNA and protein levels [91], S100A8/A9 is probably released from activated monocytes and neutrophils recruited to the site of the injury. This hypothesis is supported by an elegant study by Altwegg et al. demonstrating that in ST-elevation MI patients, S100A8/A9 is markedly increased at the site of the coronary occlusion compared to the systemic circulation [90]. Additionally, the presence of S100A8/A9-positive neutrophils and macrophages was confirmed both in the occluding thrombus and in the infarcted myocardium [90, 92]. In myocardial infarction (MI) patients, plasma S100A8/A9 levels increase before the classical markers of myocardial injury such as troponin T or creatine kinase [90] and are higher compared with patients suffering from unstable angina [87, 92]. However, S100A8/A9 is a poor diagnostic biomarker for MI in patients presenting at the emergency department with acute chest pain and does not offer additional information to the already established model based on cardiac troponin [93]. Interestingly, microarray and RT-PCR analysis of platelet mRNA revealed strikingly elevated S100A9 mRNA levels in ST-elevation MI patients compared to patients with stable angina [87]. As the platelet transcriptome is directly derived from megakaryocyte mRNA, this is likely to reflect platelet composition prior to the acute event and might be responsible for differences in platelet function between MI patients and controls. However, the presence of S100A9 mRNA in platelets has been debated by other studies [94] and it is unclear whether the activated platelets contribute to local S100A8/A9 release, as platelets in the occluding thrombus did not express the S100A8/A9 protein [90].

Compared with cardiac troponin, which is acutely released from necrotic cardiomyocytes and peaks within hours after the ischemic injury, S100A8/A9 peaks after 3–5 days and continues to be elevated for several weeks after the event [92]. S100A8/A9 levels correlate with peak white blood cell and neutrophil counts [92], possibly related to the ability of S100A8/A9 to stimulate neutrophil production in the bone marrow [49]. Human monocytes isolated from MI patients are particularly responsive to S100A8/A9-induced TLR4 upregulation and secrete increased amounts of TNF*α* and IL-6 [48, 95]. Monocyte TLR4 expression and the levels of inflammatory cytokines in plasma remain elevated for more than 14 days after the acute event and correlate with the development of heart failure [95]. These results are supported by experimental data demonstrating that TLR4 deficiency is protective against the development of cardiac dysfunction after coronary ischemia [96]. In a mouse model of coronary artery occlusion, S100A8/A9 binding to RAGE on phagocytes was shown to trigger NF-kB activation, inflammatory cytokine production, and enhanced immune cell recruitment into the myocardium [54]. Thus, S100A8/A9 amplifies the local myocardial inflammation associated with ischemia/reperfusion injury, facilitating myocardial remodeling and the development of heart failure [54].

To date, the only study assessing the value of S100A8/A9 as a potential prognostic biomarker in the immediate post-ACS period has been performed by Morrow et al. in 237 case-control pairs selected from the PROVE IT-TIMI 22 statin therapy trial cohort [97]. S100A8/A9 was measured 30 days after the acute event and found to be elevated in patients who suffered a recurrent event (MI or CV death) during the subsequent 30 day period [97]. Patients with S100A8/A9 values within the top quartile had a 2 times higher risk to develop a recurrent event compared to the lowest quartile, independently of diabetes, hypertension, previous CV disease, heart failure, and hsCRP. S100A8/A9 and hsCRP provided additive prognostic information in this population and the intensive statin therapy (atorvastatin 80 mg) lowered plasma S100A8/A9 levels compared to the moderate therapy group (pravastatin 40 mg) after 30 days of treatment [97]. S100A8/A9 was found to be increased in patients suffering from severe heart failure (NYHA class III-IV) compared to patients with hypertension or healthy subjects, in a group of elderly individuals (>70 years of age). In the heart failure group, S100A8/A9 was positively correlated with IL-6 and IL-8 and predicted all-cause mortality in 1 year [98]. However, it is unclear whether progressive heart disease was the main cause of death in this group and it remains to be determined whether S100A8/A9 is actively involved in the pathogenesis of heart failure in humans.

## 5. S100A8/A9 as Therapeutic Target

Due to its potential involvement in atherogenesis, plaque vulnerability, ischemia-associated myocardial inflammation, and heart failure, S100A8/A9 represents an attractive target in CVD. Quinoline-3-carboxamides are orally active chemical compounds with potent anti-inflammatory properties in various models of autoimmune disease such as SLE, experimental autoimmune encephalomyelitis, and collagen arthritis [99–102]. The molecular targets and therapeutic mechanisms of these compounds have initially been unknown. Recently, Björk at al. have identified S100A9 as the elusive target of quinoline-3-carboxamides [42]. The quinoline-3-carboxamide ABR-215757 binds both mouse and human S100A9 and S100A8/A9 in a Ca^2+^ and Zn^2+^ dependent manner and blocks their interaction with RAGE and TLR4 [42]. This effect is biologically relevant *in vivo*, as ABR-215757 inhibits TNF*α* production in response to LPS challenge in a mouse model, to a similar extent as a Fab antibody fragment specific for the interaction site of S100A9 with its receptors [42]. Additionally, oral ABR-215757 treatment was shown to delay disease progression in lupus-prone mice [99]. Testing quinoline-3-carboxamides as potential therapeutic principles in CVD is particularly appealing, as several of these compounds have already been approved for human use and have generated promising preliminary results in multiple sclerosis [103], juvenile type 1 diabetes [104], SLE [99], and castration-resistent prostate cancer [105]. ABR-215757 blocks S100A12 as well as S100A8/A9 and a proof-of-principle study in S100A12 transgenic hyperlipidemic ApoE^−/−^ mice demonstrated that ABR-215757 treatment reduced atherosclerotic plaque size, inflammation, and vulnerability features [106].

## 6. Conclusions and Future Directions

The experimental and clinical studies presented in the present review have demonstrated a promising potential for S100A8/A9 as a clinical biomarker and treatment target in CVD. As biomarker, S100A8/A9 correlates with the extent of subclinical carotid and coronary artery disease, increases rapidly in plasma during myocardial ischemia and necrosis, and is associated with unfavorable prognosis in MI and heart failure patients and in patients undergoing carotid arterectomy (Table 1). However, several issues remain to be elucidated before the use of S100A8/A9 can enter clinical practice. As discussed above, S100A8/A9 amplifies inflammatory processes commonly involved in the pathogenesis of atherosclerosis and autoimmune diseases. The incidence of CVD is distinctly elevated in patients with autoimmune rheumatic diseases [107] and S100A8/A9 is increased in SLE patients with CVD [89]. Prospective studies are required to determine whether S100A8/A9 measurement can offer independent information for CV risk stratification in this particular patient group. The ability of S100A8/A9 to independently predict recurrent events following an ischemic coronary event [97] needs to be compared to other biomarkers of myocardial necrosis, overload, phagocyte activation, and vascular inflammation. As the sustained inflammatory response associated with myocardial necrosis following an MI is absent in unstable angina, these patient groups should be assessed separately with regard to the prognostic value of S100A8/A9 in secondary prevention. Mouse experiments have demonstrated that S100A8/A9 is actively involved in the development of heart failure secondary to ischemia/reperfusion injury [54] and elevated S100A8/A9 concentrations are associated with increased mortality in heart failure patients [98]. Robust prospective clinical studies are required to explore whether S100A8/A9 is involved in the pathogenesis of heart failure in humans and whether plasma S100A8/A9 levels in the pre- and post-infarct period are associated with loss of cardiac function.

The main obstacle related to the use of S100A8/A9 as a therapeutic target in CVD is the relative abundance of this protein in human circulation, with median values of approximately 5 mg/L in healthy individuals, rising up to 15 mg/L in ACS patients [90, 97]. However, treatments with nontoxic doses of S100A8/A9 blockers have demonstrated encouraging results in experimental and clinical interventional studies on autoimmune disease and cancer, suggesting that complete systemic S100A8/A9 inhibition is probably not required for therapeutic effect. Topic S100A8/A9 blockade in the vulnerable atherosclerotic plaques and in the injured myocardium might provide increased efficacy and decreased systemic toxicity and represent exciting alternative approaches that need to be explored.

To conclude, S100A8/A9 seems to play a central role in the complex interactions between innate immunity, traditional CV risk factors, and CVD. Activated neutrophils and monocytes are the main sources of extracellular S100A8/A9 and diabetes, dyslipidemia, obesity, and smoking are associated with elevated circulating protein levels. S100A8/A9 seems to be involved in atherogenesis, plaque vulnerability, and post-ischemic myocardial damage. Pending further investigation, S100A8/A9 might serve as a clinical biomarker and therapeutic target in CVD, with S100A8/A9 blockers readily available and approved for clinical testing.

## Figures and Tables

**Table 1:** S100A8/A9 in cardiovascular disease.

S100A8/A9 and atherosclerosis	
Mouse studies	Present in mouse atherosclerotic plaques [71, 73]
	Reduced atherosclerotic lesions in hyperlipidemic ApoE^−/−^ S100A9^−/−^ mice [82]
	No effect on atherosclerosis in hyperlipidemic LDLR^−/−^ mice reconstituted with S100A9^−/−^ bone marrow [13]
	Reduced neointima formation in S100A9^−/−^ mice following femoral artery wire injury [82]
	Elevated plasma and plaque S100A8/A9 in diabetic ApoE^−/−^ mice [77]
Clinical studies	Present in human atherosclerotic plaques [18, 72]
	Associated with histological and ultrasound measures of plaque vulnerability [72, 85]
	Correlates with the severity of CAD in type 1 and 2 diabetic patients [49, 84]
	Correlates with carotid IMT in healthy diabetics and nondiabetics [26, 84]

S100A8/A9 in acute coronary syndrome	

Mouse studies	Accumulates into the myocardium following coronary ischemia [54]
	Triggers RAGE-mediated phagocyte activation, recruitment, and inflammatory cytokine production [54]
	Aggravates the development of post-MI heart failure [54]
Clinical studies	Increases rapidly in plasma following an ischemic coronary event [90]
	Released into the circulation from the site of the coronary occlusion [90]
	Upregulated in infiltrating neutrophils and monocytes in the infarcted myocardium and in the occluding thrombus [90, 92]
	Higher in MI patients compared to stable and unstable angina [87, 92]
	Remains elevated for several weeks after the event and correlates with peak white cell and neutrophil counts [92]

S100A8/A9 and CV risk	

Clinical studies	Correlates with short- and long-term risk for CV events in apparently healthy women independently of traditional CV risk factors [26, 87]
	Associated with the incidence of subsequent CV events in patients undergoing carotid endarterectomy [86]
	Elevated S100A8/A9 at 30 days after a coronary event is associated with increased risk for recurrent events during the following 30 day period [97]
	Associated with all-cause 1-year mortality in elderly patients with severe heart failure [98]
	Elevated in SLE patients with CV disease—retrospective study [89]
